# AIDDISON: Empowering Drug Discovery with AI/ML and CADD Tools in a Secure, Web-Based SaaS Platform

**DOI:** 10.1021/acs.jcim.3c01016

**Published:** 2023-12-22

**Authors:** Andrew Rusinko, Mohammad Rezaei, Lukas Friedrich, Hans-Peter Buchstaller, Daniel Kuhn, Ashwini Ghogare

**Affiliations:** † MilliporeSigma, 400 Summit Drive, Burlington, Massachusetts 01803, United States; ‡ Merck Healthcare KGaA, Medicinal Chemistry and Drug Design, Darmstadt 64293, Germany

## Abstract

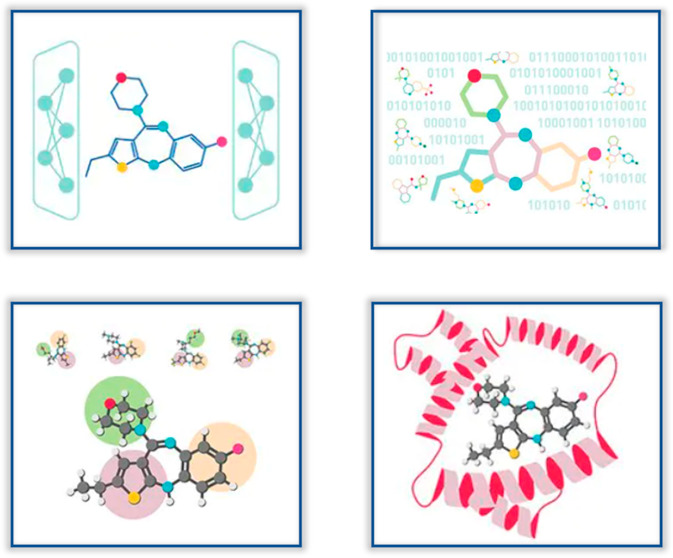

The widespread proliferation of artificial intelligence (AI) and machine learning (ML) methods has a profound effect on the drug discovery process. However, many scientists are reluctant to utilize these powerful tools due to the steep learning curve typically associated with them. AIDDISON offers a convenient, secure, web-based platform for drug discovery, addressing the reluctance of scientists to adopt AI and ML methods due to the steep learning curve. By seamlessly integrating generative models, ADMET property predictions, searches in vast chemical spaces, and molecular docking, AIDDISON provides a sophisticated platform for modern drug discovery. It enables less computer-savvy scientists to utilize these powerful tools in their daily activities, as demonstrated by an example of identifying a valuable set of molecules for lead optimization. With AIDDISON, the benefits of AI/ML in drug discovery are accessible to all.

## Introduction

The widespread popularity and use of machine learning (ML) and artificial intelligence (AI)-based methods has increased dramatically during the past decade. Applications of AI in the life sciences and specifically chemistry have seen an exponential increase in research publications since 2015.^1^ Some of the most promising areas deal with the prediction of bioactivity of novel molecules,^2^ generation of 3D protein structures from sequence data,^3^ calculation of a variety of ADME/Tox properties,^4^ as well as suggestions of synthetic routes to complex target molecules.^5^ Simultaneously, advances in virtual screening have made efficient sampling of vast chemical spaces (i.e., billions of molecules) possible,^6^ thus providing a rich source of novel and, equally important, synthesizable molecules for evaluation in ML models or docking experiments.^7^ Unfortunately, many of the recent advances are often too difficult for casual users to master since they are often deployed without a convenient and intuitive interface.

**Figure 1:**
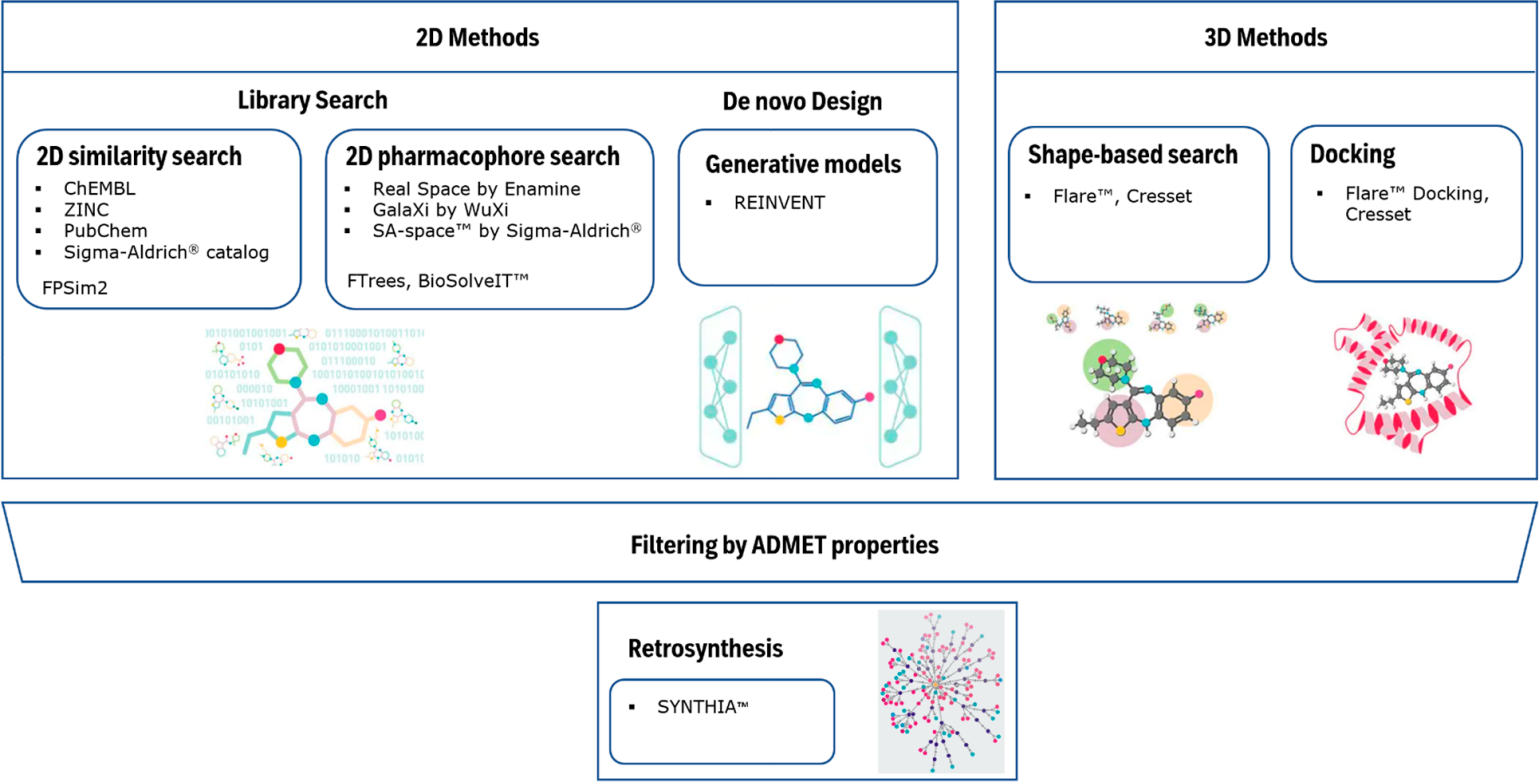
Workflows implemented in AIDDISON.

AIDDISON, an AI-powered drug discovery platform, was developed to address the need to accelerate hit identification and lead optimization in the drug discovery process by tapping into an underutilized resource—medicinal chemists themselves. Through seamless integration, AIDDISON harnesses the power of both computer-aided drug design (CADD) tools and AI to virtually screen for or generate novel molecules. As depicted in Figure 1, AIDDISON can be used to identify or generate thousands of viable molecules as the starting point in an analysis. These can come from a variety of sources—2D similarity searches of public databases, 2D pharmacophore searches of virtual chemical collections, *de novo* molecule design using generative models, and, of course, direct user input. Property-based filtering is a critical step in the process since it can be used to select those molecules to advance with the highest probability of success.^8^ Docking experiments or shape-based alignment to a known active ligand is then used to evaluate potential biological activity. Finally, the best molecular designs can be sent to SYNTHIA retrosynthesis software^5^ to assess their synthesizability and identify necessary reagents. Employing AIDDISON should enable scientists to accelerate the drug discovery process and identify novel hits faster and with better property profiles that will cause fewer failures in subsequent lead optimization and development stages. AIDDISON is based on ongoing research and active development, and a variety of functionalities are planned to be implemented in the next releases. As part of the security requirements, AIDDISON complies with ISO 27001 standards, the certificate for the highest level of information security for a digital product.

## Methods

AIDDISON was developed as a secure software as a service (SaaS) environment (following ISO 27001 standards^9^). It utilizes a cloud-based, serverless architecture, which allows companies to keep computational costs under control while giving access to proven AI as well as commercial computational technologies to end-users. All workflows are driven from a common interface, and results are displayed in a convenient, readily digestible manner.

### 2D Similarity Search

FPSim2^10^ is used to rapidly perform chemical similarity search of a given target molecule on large collections of compounds that have either been reported or are commercially available. Structural features (ECFP2) are encoded into molecular fingerprints (bitstrings) for comparison. Tanimoto similarity is then computed to the target structure. Those molecules whose similarity is above a desired threshold are returned. Currently, the available searchable collections include the Sigma-Aldrich catalog, PubChem,^11^ ChEMBL,^12^ and ZINC.^13^ Using the same search method across all databases standardizes the results.

### 2D Pharmacophore Search

The 2D pharmacophore search computes similarity to a target molecule by comparing the similarity of Feature Trees (FTrees) as implemented by BioSolveIT.^16,17^ This is a highly efficient and effective tool for scaffold hopping and ligand-based screening of incredibly vast virtual chemical space. Its underlying topological descriptors capture ring/chain and pharmacophore attributes; the relationships among these descriptors are kept intact using a reduced graph representation, thus allowing for extremely rapid comparisons.

During the past few years, the number of available screening compounds has grown larger than ever before, through both physical and virtual libraries. Billions of synthetically accessible compounds are offered by companies like Enamine (REAL space)^14^ and WuXi (GalaXi).^15^ In addition to these virtual collections, SA-Space is a synthetically accessible, ultra-large virtual chemical space generated from Sigma-Aldrich building block chemicals and well-known robust chemical transformation rules. SA-Space encompasses approximately 25 billion virtual compounds which can be searched quickly and exclusively via AIDDISON. The upcoming release of AIDDISON enables customized virtual chemical spaces based on user-supplied reactions and reagents, which can be explored using the same FTrees search algorithm.

### *De Novo* Design (Generative Models)

Recent interest in generative AI models for ligand-based *de novo* drug design has significantly increased. Within AIDDISON, *de novo* design based upon REINVENT 2.0^18^ generates a set of virtual molecules with desired chemical properties from a target molecule of interest and a desired level of chemical diversity sampled. The reward function for the reinforcement learning is composed of terms such as druglikeness (QED)^19^ and similarity to target structure (using FTrees). An additional term is used for the assessment of synthetic accessibility via a connection to SYNTHIA retrosynthesis software.^5^ We are working on extending the scoring components by user-defined models. Molecules with optimal scores are returned for further analysis. Results are evaluated, and a subset of the best are docked for evaluation of potential binding affinity.

### Shape-Based Search

**Figure 2:**
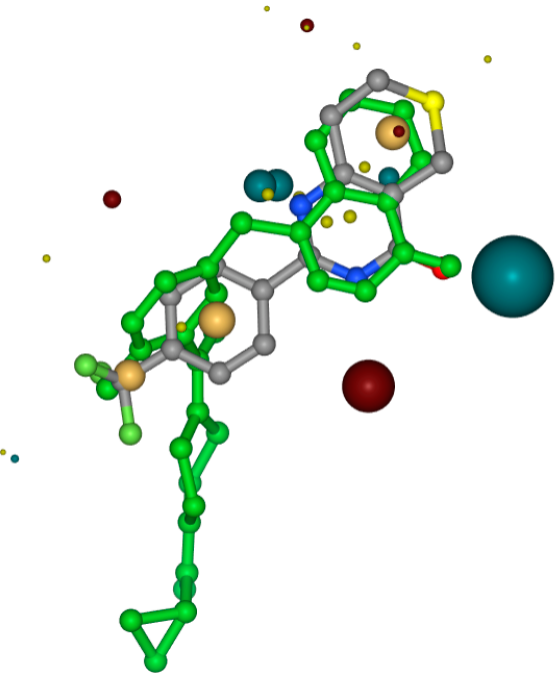
Shape-based alignment of XAV-939 against the crystal-bound conformation of Olaparib.

Shape-based search, based on Cresset’s Flare,^20^ is an effective method for evaluating novel molecules via their 3D alignment to a target molecule. Once the crystal structure or low-energy conformer has been determined for the target, property-based field points are generated for it. These field point patterns, combined with their shape, are used to align and score a “database” of molecules against a reference which is usually a known active. An example is provided in Figure 2. In this context, the default Dice similarity has worked well and provided meaningful results. The Gallery View is then used to visualize the 3D structural alignment results. Alternatively, using the asymmetric Tversky index, a user can specify whether a subfield (akin to a substructure) or superfield search is performed. For example, these can be useful in determining whether a novel bioisosteric fragment is found in a larger target molecule. Another practical use is to reduce the number of molecules sent to molecular docking by *a priori* filtering out molecules that do not align well to a reference ligand. Figure 2 illustrates the 3D shape alignment of XAV-939 against the bioactive conformation of the ligand Olaparib.^21^

### Molecular Docking

Protein structures are necessary to initiate the Molecular Docking workflow. They can be easily added to a company’s private protein collection directly through an API to the PDB^22^ and an appropriate PDB accession code. Alternatively, a user can directly upload a protein structure (in PDB format) from their own system. Druggable pockets can be identified either algorithmically, by manual specification, or from the pocket surrounding the crystal-bound ligand.^23^

Accurate modeling of protein–ligand interactions is essential for successful docking experiments. The Molecular Docking workflow, based upon Flare Docking from Cresset,^24^ is used to evaluate molecules in the binding pocket or active site of a protein. Upon selection of the target protein structure and an indication of the binding pocket (by choosing a reference ligand or pocket), hydrogens and charges are automatically added to amino acid residues (via Cresset tools). Multiple low-energy conformers are generated for the set of molecules that will be docked. The optimal pose is identified using LF RankScore which is an energy function specifically tuned to achieve a pose similar to the reported crystal ligand and then returned to the user.

**Figure 3:**
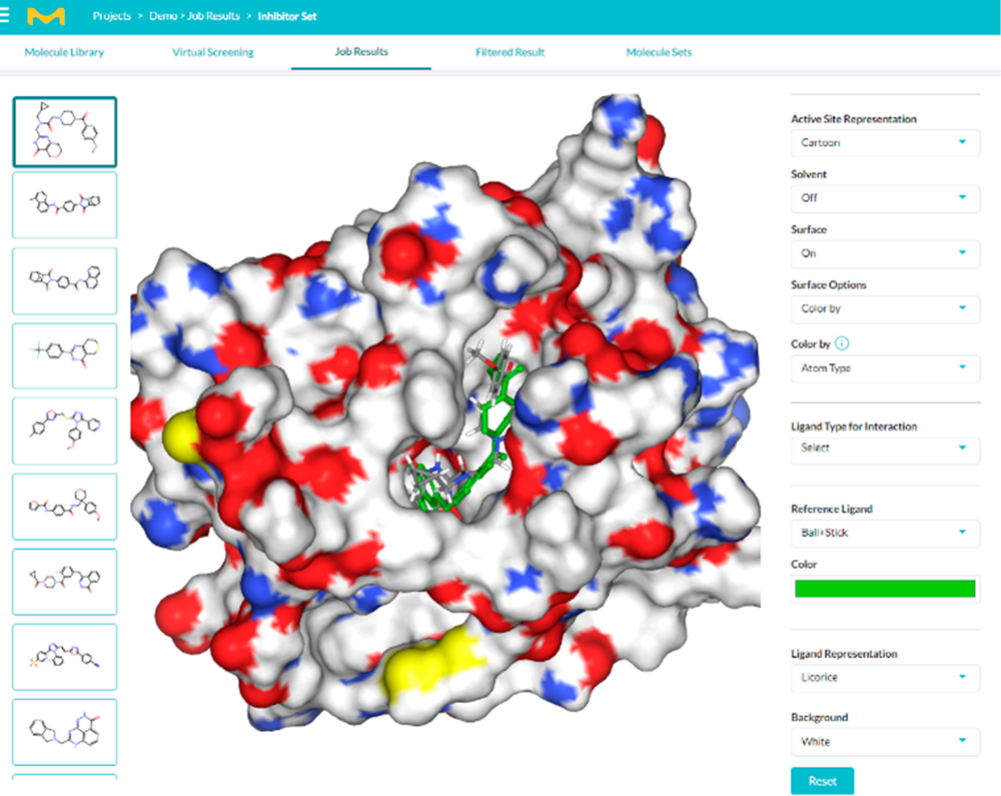
Visualization of results from Molecular Docking workflow.

Results can be visualized in a Gallery View, where the new molecules are displayed inside the chosen pocket as shown in Figure 3. The solvent-accessible surface of the protein and the reference ligand can be displayed as well. The user can thus see how each molecule “fits” in the pocket relative to the crystal structure. This also allows the user to assess the binding mode and which protein–ligand interactions are formed. A Table View gives the user a chance to sort by various computed quantities, such as VSScore (virtual screen score), which provides an indication of potential bioactivity, and a calculated docking score called “Binding Free Energy” (dG) from the Flare software. It should be noted that this is not the free energy of binding one obtains from Free Energy Perturbation (FEP) calculations. This term is comprised of the force field energy terms (enthalpy) and an estimated entropy of binding for the protein–ligand complex. Combined with calculated physicochemical properties, these energy-based parameters help to select the “best” candidates to pursue.

## Results

### Case Study. Tankyrase Inhibitors

β-Catenin is a dual function protein involved in the regulation and coordination of cell–cell adhesion, gene transcription, and overall physiological homeostasis.^25^ Mutations and overexpression of β-catenin are associated with many cancers, including hepatocellular carcinoma,^26^ colorectal carcinoma,^27^ lung cancer,^28^ malignant breast tumors,^29^ and ovarian and endometrial cancer.^30^ Though Wnt is the chief regulator of β-catenin, cellular levels are influenced by the β-catenin destruction complex (APC/GSK/CK1/Axin) which marks the protein (via phosphorylation and ubiquitination) for degradation by the proteasome. Tankyrase, on the other hand, is used by the cell to remove Axin which is a component of this complex. This limits the levels of β-catenin destruction complex and thus increases the amount of cellular β-catenin. Therefore, effectively blocking tankyrase facilitates β-catenin removal which leads to anticancer activity of tankyrase inhibitors.^31^

**Figure 4:**
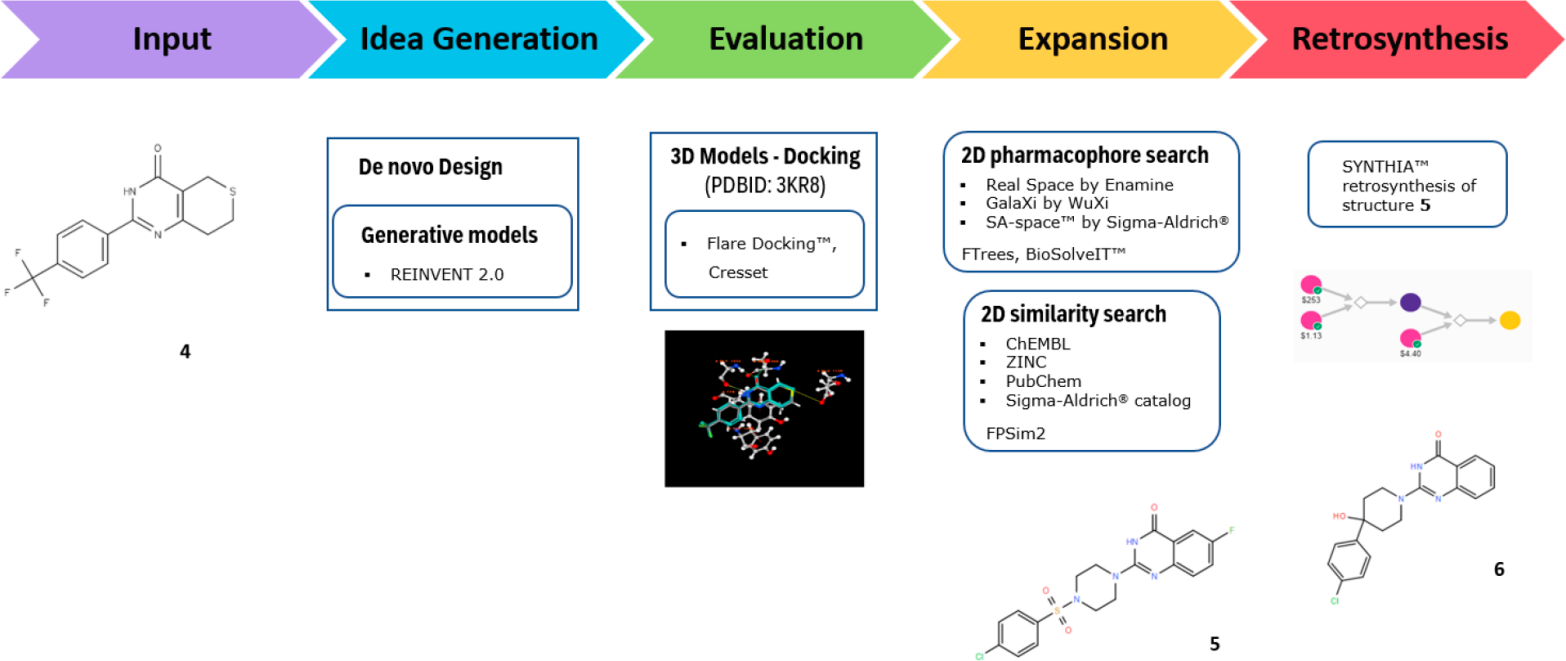
Case study: design and refinement of tankyrase inhibitors.

The challenge of discovering potentially novel tankyrase inhibitors is intriguing since many potent molecules with a variety of scaffolds are known.^32,33^ Starting from a known tankyrase inhibitor, XAV-939 (**4**)^34^ as the target, *de novo* design led to the identification of several different scaffolds which could ultimately prove to be useful. The well-known quinazoline-4(3*H*)-one scaffold (**5**) was chosen to exemplify the process. Two-dimensional similarity and pharmacophore search were used to identify a set of similar structures to construct an SAR around. The workflow is illustrated in Figure 4, where the retrosynthetic pathways were provided by SYNTHIA software.^5^ All molecules were docked using the crystal structure of XAV-939 bound to tankyrase 2 (PDBID: 3KR8).^35^ Interestingly, a known tankyrase inhibitor from ChEMBL, flavones, was identified as well from *de novo* design.^36,37^ These are shown in Table 1. In total, 14k compounds were generated using *de novo* design (2k per each of the four diversity filters based on Bemis–Murcko and topological scaffolds) and 2D pharmacophore search (6k over the three virtual chemical spaces).

**Table 1:**
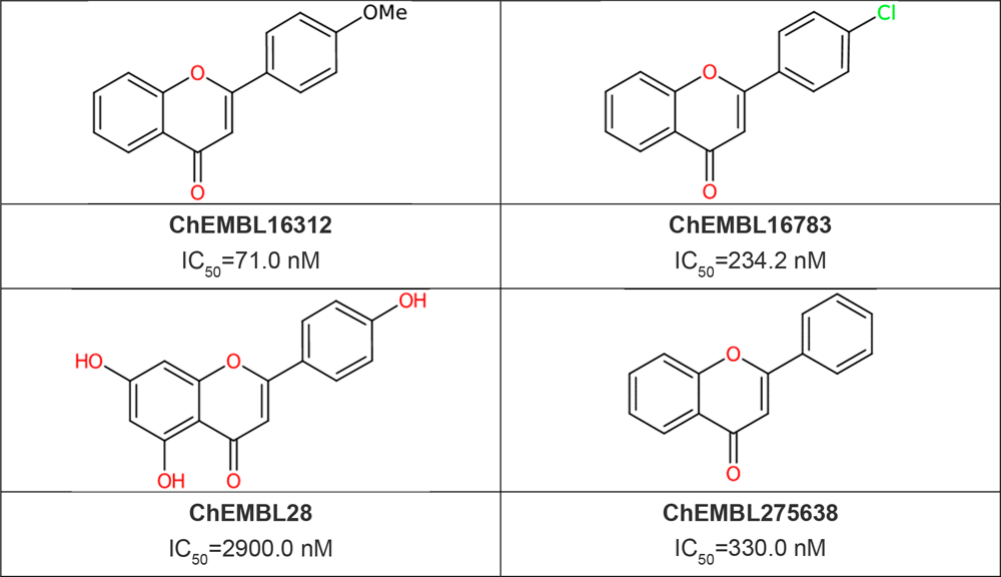
Known Tankyase Inhibitor Class (Flavones) in ChEMBL Identified from *De Novo* Design Starting from the Target Structure XAV-939 (**4**)

## Discussion

In the example provided, the process started with *de novo* design utilizing known active molecules as targets; a tankyrase inhibitor XAV-939 was used as the starting point to generate a new series of inhibitors based on the quinazoline-4(3*H*)-one scaffold.

**Figure 5:**
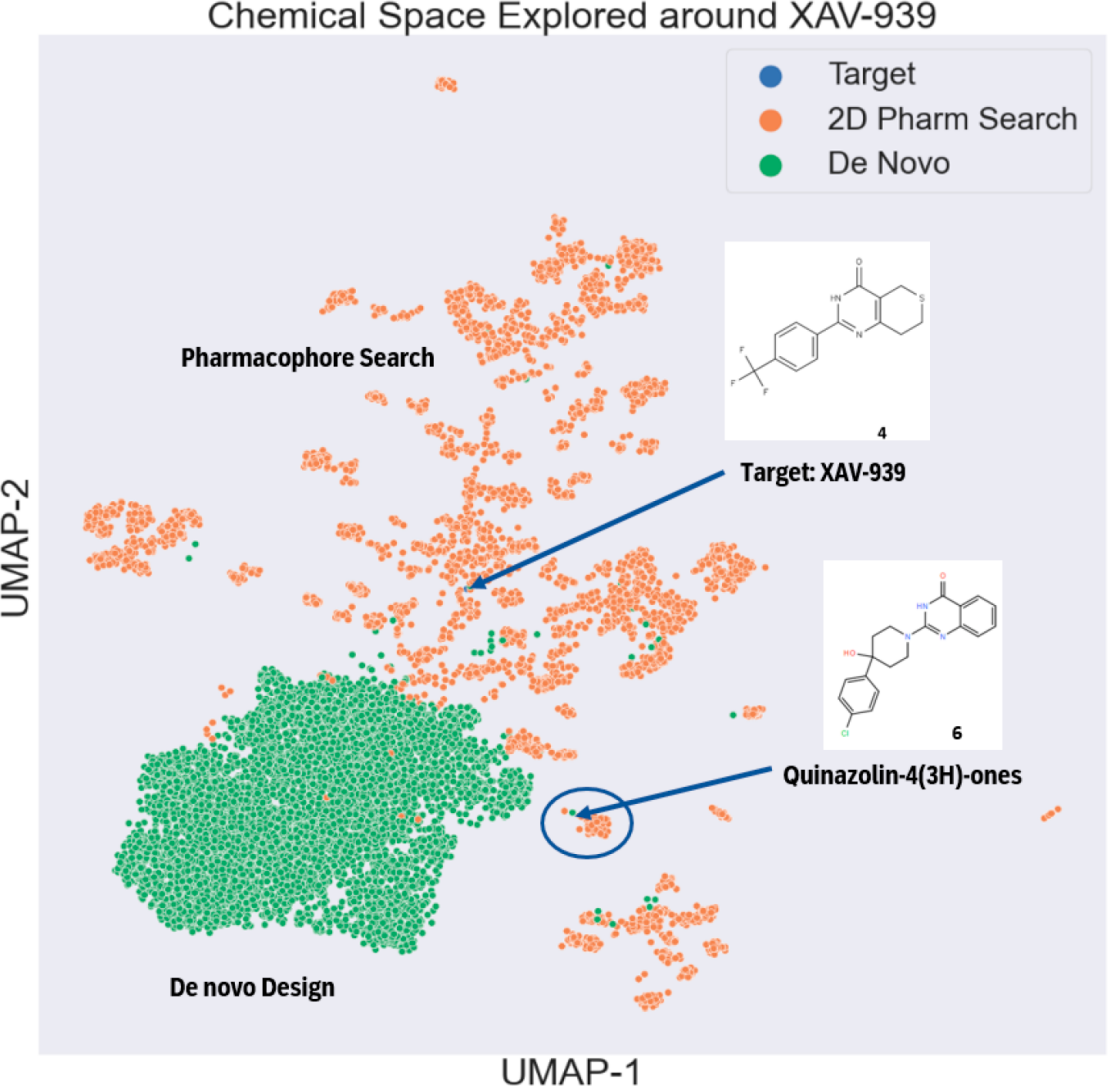
Chemical space explored around XAV-939 using *de novo* design (green) and 2D pharmacophore search (gold) of virtual collections.

The chemical space around XAV-939 (tankyrase inhibitor) was thoroughly explored by using all options of *de novo* molecule design and against all virtual compound collections. The results were first exported from AIDDISON and then uploaded and displayed by the Chemplot’s UMAP option,^38^ as shown in Figure 5. They were grouped according to structural as well as method “similarity”. The green points represent molecules that came from *de novo* molecular design while the orange points show the distribution of molecules that result from 2D pharmacophore search of virtual chemical space. The encircled region represents the quinazoline-one (**5**) from *de novo* design; it led to the identification of a set of similar molecules from 2D pharmacophore search which are predicted to be active. The distribution of both sets of molecules is reminiscent of what was illustrated by Meyers *et al*.^2^ when discussing generative models. That is, generative models thoroughly explore one region of chemical space while virtual collections sample chemical space more broadly.

It should be noted that these analyses do not have to be “one and done” in that the methods themselves are robust enough to restart with different target molecules and run multiple times if desired. The importance of screening chemical space with different methods should not be underestimated since one should not expect to find high-quality results from a single method alone. Rather, these tools are complementary, and when used in conjunction with property-based filtering of hit sets in AIDDISON, they can produce a small set of interesting molecules for synthesis and further testing.

As a note, all licenses to run commercial applications within AIDDISON are included. No additional licenses are required.

## Summary

AIDDISON is a secure, web-based SaaS drug discovery engine that combines the power of AI/ML and CADD tools in a seamless fashion. Less computationally sophisticated users can now benefit from the powerful ligand- and structure-based design tools to rapidly identify novel, potentially active molecules which have good predicted ADMET profiles and are synthetically accessible. A use case example for lead optimization was provided. The example illustrated how easy it is to generate a set of molecules for SAR analysis and lead optimization around an alternative scaffold. Reported inhibitors of tankyrase in ChEMBL were identified as well. This use-case scenario exemplifies AIDDISON capabilities and the way idea generation workflows can be used in a complementary fashion; this is an advantage AIDDISON offers.

## Data Availability

The AIDDISON platform is commercially available to the public (https://www.sigmaaldrich.com/US/en/services/software-and-digital-platforms/aiddison-ai-powered-drug-discovery). The platform incorporates REINVENT 3.2 which is publicly available from https://github.com/MolecularAI/Reinvent. The generative model is pretrained and publicly accessible from https://github.com/MolecularAI/ReinventCommunity/tree/master/notebooks/models as “random.prior.new”. The third-party software included in AIDDISON are the FTrees algorithm (v6.10) from BioSolveIT (https://www.biosolveit.de/products/#FTrees), Flare (v7.2) and pyFlare (v7) from Cresset (https://www.cresset-group.com/software/flare), and Synthia (v23.2) from MilliporeSigma, the U.S. and Canada Life Science business of Merck KGaA, Darmstadt, Germany (https://www.synthiaonline.com). All physicochemical properties and the clustering algorithms were implemented using RDKit (https://github.com/rdkit/rdkit). The starting molecules, the resulting compounds, and the parameters to run the workflows in the case study are shared at Zenodo (https://zenodo.org/record/10231008).
